# 2023 Reviewer Index

**DOI:** 10.26508/lsa.202402576

**Published:** 2024-01-16

**Authors:** Eric Sawey

**Affiliations:** Executive Editor, Life Science Alliance, Cold Spring Harbor, NY, USA

## Abstract

The LSA Editors thank these scientists for sharing their time and expertise while reviewing manuscripts in 2023. We sincerely appreciate their support.

Aachoui, Youssef

Aavula, Kumar

Abankwa, Daniel

Abdel-Wahab, Omar

Abel, Steven

Acharya, Priyamvada

Adams, Josephine C.

Adhikari, Deepak

Adhikari, Hema

Agallou, Maria

Aguirre, Aitor

Ahn, Natalie G.

Aidinis, Vassilis

Akat, Kemal M.

Akay, Alper

Akondy, Rama S.

Al-Bataineh, Mohammad Mahmmoud

Alarcon, Balbino

Alder, Louise

Alexander, Matthew

Alhafi, Anas

Allalou, Amin

Allen, Lee-Ann H.

Allred, David R.

Almeida, Miguel

Altintas, Dogus M.

Amberg, Nicole

Anand, Paras K.

Anjo, Sandra I.

Ansa-Addo, Ephraim A.

Antonescu, Costin N.

Antonin, Wolfram

Arndt, Karen M.

Aronow, Bruce J.

Arora, Kavisha

Arpağ, Göker

Aryal, Uma

Asim, Mohammad

Assis, Castro D.

Autexier, Chantal

Azhar, Mohamad

Aztekin, Can

Bénard, Giovanni

Böhm, Volker

Böttcher, Ralph T.

Böttcher-Friebertshäuser, Eva

Babu, Mohan

Baes, Myriam

Baeza Garcia, Alvaro

Baffet, Alexandre

Bain, Calum C.

Baker, Kristian E.

Bakkenist, Christopher J.

Balmus, Gabriel

Baltz, Jay

Ban, Young H.

Bano, Daniele

Barbazán, Jorge

Baritaki, Stavroula

Barrantes, Francisco J.

Bartek, Jiri

Barton, Michelle (Shelly) C.

Bashaw, Greg J.

Bastepe, Murat

Baugh, Ryan

Baulac, Stephanie

Baumgarth, Nicole

Bayne, Elizabeth

Becker, Peter B.

Becker, Thomas

Becker, Walter

Beers, Stephen A.

Belser, Jessica

Benavente, Claudia A.

Benner, Christopher

Berezin, Mikhail

Bergeron, John J.

Bergmann, Andreas

Berthelot, Camille

Bertolin, Giulia

Bhattacharyya, Suvendra N.

Bhavsar, Swapnil

Bi, Erfei

Bi, Pengpeng

Biryukova, Inna

Blüthgen, Nils

Blacklow, Stephen C.

Blouin, Cedric

Blount, Paul

Blum, Robert

Bonilha, Vera L.

Bonner-Weir, Susan

Bonnet, Dominique

Borggaard, Xenia Goldberg

Borin, Veniamin A.

Boucher, Dave

Bourdin, Arnaud

Bournazos, Stylianos

Bowerman, Melissa

Brabek, Jan

Bradke, Frank

Braid, Lorena R

Brasselet, Sophie

Braun, Ralf J.

Braun, Sigurd

Breunig, Joshua J.

Brody, Jonathan

Brookes, Emily

Brown, Myles

Brul, Stanley

Brunelli, Silvia

Bruns, Heiko

Bryder, David

Buchberger, Alexander

Bullock, Simon L.

Busch, Karin B.

Bussolino, Federico

Calderon de Anda, Froylan

Campaner, Stefano

Campbell, Kirk

Carrasco, Elisa

Carrer, Alessandro

Carrier, France

Carro, Maria Stella

Carter, Andrew P.

Carvalho, Claudia

Carver, Brett S.

Caspary, Tamara

Castren, Eero

Chacinska, Agnieszka

Chai, Zuying

Chakrabarty, Yogaditya

Chanfreau, Guillaume

Chang, Eric C.

Chappe, Valerie M.

Chassen, Stephanie

Chatterjee, Nimrat

Chen, Bill B.

Chen, Haide

Chen, Jiekai

Chen, John

Chen, Jun

Chen, Lingling

Chen, Lu

Chen, Sheng-hong

Cho, Hee C.

Chong, Shasha

Chrzanowska-Lightowlers, Zofia M.

Chuva de Sousa Lopes, Susana M.

Cingaram, Pradeep

Cipollina, Chiara

Clague, Michael J.

Clyne, Alisa M.

Cobine, Paul

Cogné, Michel

Collao, Nicolas

Colque, Antonella

Columbano, Amedeo

Compton, Alex A.

Conboy, John G.

Conde, Carlos

Contreras, Osvaldo

Conway, James

Cook, Leah

Copeland, William C.

Cordat, Emanuelle

Cordes, Thekla

Costanzo, Vincenzo

Cougnoux, Antony

Courchet, Julien

Cox, Timothy

Crespi, Martin

Crichton, Paul G.

Cui, Rutao

Cvetanovic, Marija

Dahlin, Joakim

Dalal, Yamini P.

Daly, Ann

Damm, Georg

Dantuma, Nico P.

Dantzer, Françoise

Das, Rudra N.

Das, Sasti Gopal

Dash, Rupesh

Davie, Judith K.

Davies, Clare C.

Davuluri, Ramana V

Day, Catherine L.

De Almeida, Rodrigo F.

De Juan-Sanz, Jaime

Debyser, Zeger

D oly, Etienne

Defossez, Pierre-Antoine

Degterev, Alexei

Del Río Serrato, Alba

Delvendahl, Igor

Demeulemeester, Jonas

Den Brave, Fabian

Deng, Qiaolin

Desai, Arshad

Deshpande, Nikita

Desingu Rajan, Ayyappa Raja

Després, Philippe

Dettmer, Ulf

Dewson, Grant

Dhillon, Navneet

Dick, Fred A.

Dickman, Dion

Dietrich, Alexander

Dikstein, Rivka

Dimarco, Federico

Dimitriadi, Maria

Dimitrov, Jordan D.

Dineley, Kelly T.

Ding, JunJun

Dittmann, Meike

Diz-Muñoz, Alba

Djian, Philippe

Djiane, Alexandre

Dohmen, R. Jürgen

Doitsidou, Maria

Dolezal, Pavel

Dominguez, Cecilia

Dong, Zheng

Dongari-Bagtzoglou, Anna

Downward, Julian

Draviam, Viji

Drosten, Matthias

Ducret, Axel

Dudanova, Irina

Duffy, Diane

Dykhuizen, Emily C.

Dyson, Jennifer M.

Dzierzak, Elaine

E, Lezi

Ebert, Peter

Eeckhoute, Jerome

Eliceiri, Brian P.

Erlacher, Matthias

Ernst, Patricia

Escalante, Ricardo

Estella, Carlos

Extavour, Cassandra

Eyers, Patrick A.

Fandrey, Joachim

Farber, Steven

Farhan, Hesso

Farinha, Carlos M.

Fei, Ji-Feng

Ferarrio, Carlos

Fernandez-Chacon, Rafael

Fernandez-Mariño, Ana I.

Fernandez-Zapico, Martin E.

Fernie, Alisdair R.

Ferrer, Jorge

Figiel, Maciej

Firon, Arnaud

Fisher, Robert P.

Fodde, Riccardo

Foley, Ann

Folsch, Heike

Fon, Edward A.

Fontanesi, Flavia

Foulkes, Nicholas

Fourati, Slim

Fournier, Marjorie

Franken, Gijs

Fraser, Alasdair R.

Frazer, Kelly A.

Friedman, Jonathan

Froehlich, Dominik

Fromm, Michael

Fromme, J. C.

Fu, Hailu

Fu, Rui

Götz, Magdalena

Gümüşoğlu, Serena

Gabrielli, Brian G.

Gaines, Peter

Galande, Sanjeev

Gale, Daniel

Gallagher, Thomas

Gallouzi, Imed E.

Galupa, Rafael

Gammoh, Noor

Gangurde, Suni S

Garabedian, Michael

Garapati, Kishore

Garcia, Sarela

Garcia-Mata, Rafael

Gardella, Thomas

Garlanda, Cecilia

Gau, David

Ge, Liang

Gebauer, Fatima

Geijtenbeek, Teunis B.

Gentzsch, Martina

Georgiades, Emily

Gernaey, Krist

Gersch, Malte

Ghaffari, Saghi

Giamas, Georgios

Gilkerson, Robert

Gillet, Reynald

Gingras, Marie-Claude

Giron, Jorge A.

Giurisato, Emanuele

Gladkova, Christina

Goke, Jonathan

Goldberg, Alfred L.

Gombert, Andreas

Gomez-Lopez, Nardhy

Gorsope, Myriam

Goto-Inoue, Naoko

Gotta, Monica

Goud, Bruno

Gough, Michael J.

Graef, Martin

Grant, Struan F.

Grencis, Richard K.

Grillet, Nicolas

Grochowski, Christopher

Gross, Alecia K.

Gross, Atan

Gruber, Stephan

Grumati, Paolo

Grunwald, Gerald B.

Gu, Xinliang

Gugasyan, Raffi

Gumz, Michelle

Gunning, Peter W.

Gursky, Jan

Gutschner, Tony

Gwack, Yousang

Gylys, Karen H.

Haber, James E.

Habib, Amyn A.

Haesemeyer, Martin

Haeusler, Aaron

Hahn, Bevra

Halazonetis, Thanos D.

Halberg, Nils

Haltalli, Myriam

Hamada, Mayuko

Hamilton, Brett

Hammond, Gerald R.

Han, Arnold

Hanigan, Thomas

Hannon, Eilis

Hansen, Scott B.

Hantke, Klaus

Hao, Bingtao

Hardikar, Swanand

Harel, Amnon

Hashimoto, Hiroshi

Hattori, Motoyuki

Hawinkels, Lukas J.

He, Bai-Cheng

He, Sicong

He, Yong

Heineke, Joerg

Heinisch, Jürgen J.

Hejatko, Jan

Helft, Julie

Helgason, Vignir

Hemberger, Myriam

Hendricks, Adam G.

Herault, Yann

Herbert, DeBroski R.

Herker, Eva

Hermey, Guido

Hernandez, Greco

Herrmann, Christian

Herrmann, Johannes M.

Hesselink, Matthijs K.

Hiemstra, Pieter S.

Higashi, Tomohito

Hinnebusch, Alan G.

Hino, Shinjiro

Hirrlinger, Johannes

Hnia, Karim

Hochstrasser, Mark

Hodson, Daniel J

Hogg, Robert

Holloman, William K.

Horii, Mariko

Horovitz, Amnon

Hou, Xu

Hsiao, Kuei-Yang

Hu, Qi

Huang, Dongsheng

Huang, Jean

Huang, Lan

Hudson, Matthew

Humphrey, Mary B.

Huynh, Jean-Rene

Ichinose, Hiroshi

Ichtchenko, Konstantin

Igreja, Catia

Ihrie, Rebecca A.

Ilkayeva, Olga

Inaba, Kazuo

Innocenti, Metello

Iorio, Francesco

Irigoin, Florencia

Ito, Toshihiro

Jaiswal, Jyoti

Jansen, Gerrit

Janssen-Heininger, Yvonne

Jaune-Pons, Emilie

Javed, Elham

Jeannotte, Lucie

Jebet, Audriy

Jenq, Robert R.

Jensen, Uffe B.

Jessberger, Sebastian

Jhunjhunwala, Suchit

Ji, Peifeng

Jiang, Hui

Jiang, Yu-Yang

Jiao, Kai

Jimenez, Connie R.

Joazeiro, Claudio A.

Jochmans, Dirk

Johansen, Terje

Johnson, Aaron

Johnson, Daniel M.

Johnson, Lance

Johnstone, Scott

Jones, David K.

Judge, Andrew R

Kabani, Mehdi

Kabir, Ashraf Ul

Kadin, Marshall E.

Kakumani, Pavan K.

Kandoi, Sangeetha

Kapranov, Philipp

Kapus, Andras

Karginov, Andrei V.

Karner, Courtney M.

Karppinen, Peppi

Karwi, Qutuba

Kashani, Amir

Katz, David J.

Kee, Kehkooi

Kermorgant, Stephanie

Ketting, Rene F.

Khalimonchuk, Oleh

Khan, Kasim

Khan, Muzamil

Khan, Wasim

Khanna, Hemant

Khetani, Salman R.

Kim, Jun Yong

Kimpel, Janine

King, Cecile

Kinsey, William

Kläsener, Kathrin A.

Kleinridders, Andre

Klemm, Robin

Klymkowsky, Michael

Kohwi-Shigematsu, Terumi

Kolch, Walter

Kolmogorov, Mikhail

Komatsu, Yoshihiro

Kong, Daochun

Konno, Tasuku

Korotkov, Konstantin V.

Kouzine, Fedor

Kovall, Rhett A.

Kröger, Andrea

Kraft, Mary

Krauss, Michael

Kravats, Andrea

Kulms, Dagmar

Kumar, Vinod

Kunkel, Thomas A.

Kyrousi, Christina

Lachmann, Nico

Lai, Xianyin

LaMarca, Babette D.

Langer, Thomas

Laqtom, Nouf

Lareau, Caleb

LaRock, Christopher N.

Lasecka-Dykes, Lidia

LaSpada, Albert

Laursen, Willem J.

Lavelle, Ed C.

Lazartigues, Eric

Le Riche, Alizee

Lee, Caroline G.

Lee, Cheol-Koo

Lee, Teresa

Lees-Miller, Susan P.

Leichert, Lars I.

Leng, Roger P.

Lesch, Bluma

Levi, Benjamin

Levin, David E.

Li, Chia-Wei

Li, Jianjian

Li, Qianjin

Liccardi, Gianmaria

Liesa, Marc

Lieske, John

Lightowlers, Robert N.

Lin, Chen-Ching

Lin, Chih-Wei

Lin, Shih-Ming

Littleton, J. T.

Liu, Bao

Liu, Feng

Liu, Ji-Long

Liu, Jingze

Liu, Wanlu

Loeb, Lawrence

Long, David A.

Lopez-Mosqueda, Jaime

Lou, Emil

Lozach, Pierre-Yves

Lu, Tao

Luan, Yi

Lugli, Gabriele Andrea

Lusis, Aldons J.

Lynn, Melissa L.

Ma, Li-Tian

Ma, Liang

Machida, Yuichi J.

Maharana, Shovamayee

Maire, Pascal

Maizel, Alexis

Makeyev, Eugene V.

Malamud, Mariano

Malhotra, Vivek

Mali, Girish

Mammucari, Cristina

Mancilla-Galindo, Javier

Mansky, Kim

Mansour, Marc R.

Marasca, Federica

Marashi, Sayed Amir

Marfany, Gemma

Mark, Brian L.

Marsili, Luca

Martin, James F.

Martinez, Aitor

Martinez-Morales, Juan R.

Marygold, Steven

Mashek, Doug

Mastrianni, James

Matassa, Danilo S.

Matteoli, Gianluca

Mattick, John S.

Maurange, Cedric

Mayor, Satyajit

McCall, Kim

McCann, Chase

McCarty, Nael A.

McCulloch, Chris A.

McDermott, Ultan

McInerney, James

McIntyre, Thomas M.

Medin, Jeffrey A.

Meier, Kathryn

Meisinger, Chris

Meloni, Valeria

Mendillo, Marc L.

Meylan, Etienne

Miceikaite, Ieva

Michaud, Morgane

Michod, David

Miller, Brandon A

Miroux, Bruno

Mishina, Yuji

Mittnacht, Sibylle

Miyazawa, Keiji

Modrich, Paul

Moffat, Jason

Moffitt, Andrea

Mogk, Axel

Mole, Sara E.

Montag, Judith

Moon, Jee-young

Moore, Kathryn

Moos, Torben

Morfini, Gerardo A.

Moroy, Tarik

Mortellaro, Alessandra

Motorin, Yuri

Mott, Helen

Mouneimne, Ghassan M.

Mounier, Rémi

Moustakas, Aristidis

Mueller, Jacob

Murphy, Phil

Musselman, Catherine

Mylavarapu, Sivaram V.

Nagl, Markus

Naiman, Karel

Nakanishi, Makoto

Nakano, Hiroyasu

Nakayama, Jun-ichi

Nakayama, Keiko

Namba, Takashi

Napierala, Marek

Naquet, Philippe

Narbonne, Patrick

Naren, Anjaparavanda P.

Nasmyth, Kim A.

Nasser, Waseem

Navaratnarajah, Chanakha K.

Neale, Matt J.

Negri, Rodolfo

Neiman, Aaron M.

Nellist, Mark

Neri, Dario

Nesvick, Cody

Neubauer, Peter

Newton, Robert

Nielsen, Anders Lade

Niemi, Natalie M.

Nikolova, Olga

Nixon, Ralph A.

Noh, Kyung-Min

Nordlund, Jessica

Norris, Adam

Notterpek, Lucia

Novershtern, Noa

Nuessler, Andreas

Nussinov, Ruth

Oda, Yoshihisa

Ohta, Akio

Olafsson, Gudjon

Oliveira, Raquel A.

Omar, Shatha

Onteru, Suneel

Oshiumi, Hiroyuki

Ott, Jurg

Ou, Xijun

Owens, Nick

Ozisik, Ozan

Pönisch, Wolfram

Paavilainen, Ville O.

Pagliarini, David J.

Pai, Athma A.

Pal, Utpal

Parchure, Anup

Park, Miso

Paronetto, Maria Paola P.

Patil, Kiran

Patra, Krushna

Patras, Kathryn

Patrignani, Paola

Payne, Elspeth M.

Pearce, Edward J.

Pearring, Jillian N.

Pearson, Melanie

Peled, Jonathan U.

Pellman, David S.

Peltzer, Nieves

Perecin, Felipe

Pereira, Joao P.

Perez, Franck

Pessler, Frank

Petsalaki, Evangelia

Pfeffer, Suzanne R.

Philips, Mark R.

Pichierri, Pietro

Pichlmair, Andreas

Pigazzi, Martina

Pillai, Ramesh S.

Pilz, Gregor

Pina, Cristina

Pinaud, Eric

Pinilla, Estéfano

Pisani, Francesca M.

Pizzo, Salvatore

Plosch, T

Poelzing, Steven

Poirier, Guy

Polacek, Norbert

Pollard, Luther

Port, J. D.

Portugal, Silvia

Powell, Jennifer

Powell, Joseph

Powell, Simon N.

Prajapati, Hemant

Prakash, Hridayesh

Prat, Alexandre

Prekeris, Rytis

Prieto-Echagüe, Victoria

Proschel, Christoph

Proux-Gillardeaux, Véronique

Pucéat, Michel

Pugacheva, Elena

Pujol, Aurora

Pulman, Juliette

Puri, Pier L.

Putnam, Chris

Pyle, Anna M.

Quax, Wim

Ríos, Rosa M.

Rabinowitz, Joshua

Rai, Asit

Raine, Amanda

Raj, Bushra

Rajagopalan, Ramakrishnan

Ralser, Markus

Ramachandran, Srinivas

Ramamurthy, Visvanathan

Raman, Malavika

Rampelt, Heike

Ran, Sophia

Ranadheera, Charlene

Rao, Ashish

Raote, Ishier

Rast, Jonathan

Rees, Elliott

Reichmann, Dana

Rein, Alan

Remenyi, Attila

Reyes Palomares, Armando

Richards, Joanne

Riley, Paul R.

Rios-Barrera, Luis D.

Ritchie, Matthew E.

Ritchie, Scott

Rittinger, Katrin

Robert, Francois

Roche, Serge

Rodriguez-Vita, Juan

Rohn, Jennifer L.

Rokosh, Gregg

Romanin, Christoph

Rosina, Marco

Rotblat, Barak

Rothenberg, Ellen V.

Rout, Michael P.

Roux, Kyle J.

Rubinstein, Eric

Rubinstein, Menachem

Rubio, Ignacio

Rudolph, Karl-Lenhard L.

Rushworth, Stuart A.

Russo, Brian

Ruthazer, Edward S.

Ryan, Dylan

Ryan, Michael T.

Ryoo, Hyung Don

Sánchez-Madrid, Francisco

Saada-Reisch, Ann

Sablina, Anna A.

Sadoshima, Junichi

Sadowski, Martin

Saharinen, Pipsa

Sahin, Umut

Sahu, Parul

Saiardi, Adolfo

Sakai, Yasuyoshi

Salzer, Ulrich

Sanders, Justin

Sandoval, Wendy

Sanjay, Archana

Sansom, David M.

Sansom, Owen

Santos-Rebouças, Cíntia B

Sareila, Outi

Sargurupremraj, Murali

Sarkisian, Matthew R.

Sarnataro, Daniela

Sasca, Daniel

Savignac, Magali

Savino, Aurora

Saxena, Smita

Scaglione, Matt

Scheuermann, Richard

Schick, Sandra

Schiebel, Elmar

Schlegel, Amnon

Schloer, Sebastian

Schneider, Günter

Schuijers, Jurian

Schwartz, Laura L.

Schwartz, Schraga

Schworer, Stephen

Seger, Rony

Senavirathna, Indika

Sengupta, Abhishek

Serin-Harmanci, Akdes

Seydoux, Geraldine

Shah, Girish M.

Shahbazi, Marta

Shao, Jian-Zhong Z.

Sharma, Abhay

Shelburne, Samuel A.

Sheltzer, Jason

Shen, Cailiang

Shi, Hao

Shi, Jia-Xin

Shi, Xiaobing

Shimada, Hiroyuki

Shimada, Issei S.

Shin, Donghyuk

Shinbrot, Eve

Shinohara, Akira

Shiraishi, Yuichi

Shirinian, Margret

Shore, Eileen

Shroff, Ankit

Shukla, Vinay

Sibon, Ody C.

Siemens, Jan

Silva, Aristobolo M.

Silva, Jose

Silvin, Aymeric

Simmen, Thomas

Simmons, James A

Sivak, Jeremy M.

Slonchak, A

Smith, Adam

Smogorzewska, Agata

Smythe, Elizabeth

Snyder, Elizabeth

Snyder, Nathaniel W.

Sobota, Radoslaw

Sokhansanj, Bahrad A

Solari, Florence

Soller, Matthias

Sonenberg, Nahum

Speck, Christian

Spivakov, Mikhail

Spletter, Maria L.

Srivastava, Pramod K.

Stainier, Didier Y.

Stefan, Christopher J.

Stojic, Lovorka

Strober, Warren

Su, Tin T.

Su, Wenru

Suda, Toshio

Suetsugu, Shiro

Sun, Chong

Sun, Hui

Sun, Xiaofei

Sun, Xueqin

Sun, Yang

Sun, Yuanye

Sung, Li-Ying

Sung, Sibum

Sutandy, FX R.

Svitkina, Tatyana M.

Svoboda, Petr

Swanson, Chad M.

Szczepanowska, Karolina

Takeda, Makoto

Takeuchi, Osamu

Tan, Aimee

Tan, Xiao-Di

Tanaka, Kozo

Tantin, Dean

Tartare-Deckert, Sophie

Tartour, Eric

Techer, Hervé

Teif, Vladimir B.

Telange, Rahul

Tellier, Michael

Tenbrock, Klaus

Teschendorff, Andrew

Thannickal, Victor J.

Thijssen, Rachel

Tikhanovich, Irina

Tomasetto, Catherine L.

Torrent, Marc

Tosches, Maria

Toshchakov, Vladimir Y.

Toulis, Vasileios

Townson, David

Trehanpati, Nirupma

Tremblay, Marie-Eve

Trempe, Jean-Francois

Tripathi, Subhash

Trotti, Davide

Truant, Ray

Tsai, Feng-ching

Tsuboi, Takafumi

Tsubouchi, Hideo

Tu, Bo

Turcan, Şevin

Turgay, Kürsad

Turner, Tychele

Tyson, Darren R.

Tzahor, Eldad

Tzfati, Yehuda

Ugolini, Camilla

Uhlirova, Mirka

Ulitsky, Igor

Umasankar, Perunthottathu K.

Umer, Husen M.

Uribe, Mary Luz

Ushakov, Dmitry S.

Uyeda, Taro

Vaandrager, Bas

Valdmanis, Paul N.

Vamosi, Gyoergy

Van Acker, Zoë

Van De Weghe, Julie C.

Van den Ameele, Jelle

Van den Boom, Johannes

Van der Does, Anne

Van Doren, Mark B.

Van Haaften, Gijs

Van Strijp, Jos A.

Vance, Keith W.

Veler, Hana

Verma, Navin Kumar K.

Verne, Nicholas

Veyrier, Frederic J.

Vicent, Guillermo P.

Villeneuve, Julien

Virshup, David M.

Viscomi, Carlo

Vogel, Kevin

Voisinne, Guillaume

Von Heijne, Gunnar

Vucic, Domagoj

Wahl, Markus

Waisman, Ari

Walko, Gernot

Walter, Matthew

Wan, Xuan (Olivia)

Wang, Hai

Wang, Hu

Wang, Ruo

Wang, Tianyi

Wang, Xinquan

Wang, Xiu-Jie

Wang, Yidong

Wang, Yuexiang

Wang, Zhong

Warren, Alan J.

Washburn, Michael

Watson, Emma

Weaver, Benjamin

Wedegaertner, Philip B.

Wei, Pei-Chi

Weidberg, Hilla

Weijers, Dolf D.

Wellstein, Anton

Welner, Rob

Wen, Wei

Werner, Achim

West, Ryan J. H.

Wiener, Reuven

Wiley, H. S.

Wilk, Sherwin

Williams, Anna

Williams, Joanna

Wilmanns, Matthias

Wilson, Danny W.

Wilusz, Jeffrey

Winklhofer, Konstanze F.

Wiseman, R L.

Wittig, Burghardt

Wohlever, Matthew

Wolf, Diana

Wong, Ping-Pui

Wu, Mingfu

Xia, Huiming

Xiao, Tianshu

Xiao, Tsan S.

Xiao, Zhan

Xiaofeng, Dong

Xu, Chenling

Xu, Guoliang

Xu, Guotai

Xu, Mengyang

Xu, Xufeng

Yélamos, José

Yamamoto, Hayashi

Yamamoto, Masahiro

Yan, Shan

Yan, Shun

Yao, Changfu

Yin, Shen

Yin, Shiyi

Ying, Zheng

Yokota, Takafumi

Yoneyama, Mitsutoshi

Yoo, Joo-Yeon

Yoshida, Hiderou

Yu, Bo

Yu, Edward W.

Yu, Luyang

Zanin, Esther

Zeng, Fuxing

Zeviani, Massimo

Zhang, Jian

Zhang, Yong

Zhang, Yunxiao

Zhang, Zhiping

Zhao, Quan

Zheng, Wang

Zhou, Xiaobo

Zhou, Yong

Zhou, Zhongjun

Zhu, Lisha

Zoladek, Teresa

